# Factors impacting antenatal care utilization: a systematic review of 37 fragile and conflict-affected situations

**DOI:** 10.1186/s13031-022-00459-9

**Published:** 2022-06-11

**Authors:** Kameela Miriam Alibhai, Bianca R. Ziegler, Louise Meddings, Evans Batung, Isaac Luginaah

**Affiliations:** 1 Faculty of Medicine, University of Ottawa, 451 Smyth Road, Ottawa, ON K1H 8M5 Canada; 2 DeGroote School of Medicine, McMaster University, 1280 Main Street W, Hamilton, ON L8S 4L8 Canada; 3 Department of Geography, Western University, 1151 Richmond Street, London, ON N6A 3K7 Canada; 4 Environment Health and Hazards Lab, Western University, 1151 Richmond Street, London, ON N6A 3K7 Canada

**Keywords:** Fragile and conflict-affected situations, Antenatal care, Maternal health, Maternal mortality, Sustainable development goals

## Abstract

**Background:**

It is estimated that over 930 million people live in fragile and conflict-affected situations (FCAS) worldwide. These regions, characterized by violence, civil unrest, and war, are often governed by corrupt administrations who are unwilling to provide their citizens with basic human rights. Individuals living in FCAS face health inequities; however, women are disproportionally affected and face additional barriers to accessing sexual and reproductive services, including antenatal care (ANC). This systematic review aims to identify the factors that impact ANC usage in the 37 countries or regions classified as FCAS in 2020 by The World Bank.

**Methods:**

Using the PRISMA guidelines, a systematic search of five databases (SCOPUS, Web of Science, PubMed, EMBASE, and CINAHL) was conducted. Results were limited to human studies, written in English, and published between January 2002 and January 2022. Studies that identified factors affecting utilization of ANC or maternal health services were included for review and critically appraised using the National Institute of Health’s Quality Assessment Tools. Findings were summarized using a narrative synthesis approach.

**Results:**

The database search yielded 26,527 studies. After title, abstract and full-text review, and exclusion of duplicate articles, 121 studies remained. Twenty-eight of the 37 FCAS were represented in the included studies. The studies highlighted that women in FCAS’ are still not meeting the World Health Organization’s 2002 recommendation of four ANC visits during pregnancy, a recommendation which has since been increased to eight visits. The most cited factors impacting ANC were socioeconomic status, education, and poor quality of ANC. Despite all studies being conducted in conflict-affected regions, only nine studies explicitly identified conflict as a direct barrier to accessing ANC.

**Conclusion:**

This review demonstrated that there is a paucity in the literature examining the direct and indirect impacts of conflict on ANC utilization. Specifically, research should be conducted in the nine FCAS that are not currently represented in the literature. To mitigate the barriers that prevent utilization of maternal health services identified in this review, policy makers, women utilizing ANC, and global organizations should attempt to collaborate to enact policy change at the local level.

**Supplementary Information:**

The online version contains supplementary material available at 10.1186/s13031-022-00459-9.

## Introduction

As of 2022, it is estimated that over 930 million people live in fragile and conflict-affected situations (FCAS) worldwide and the number of individuals affected by conflict continues to rise [1]. FCAS are countries or regions characterized by a high propensity for recurring conflict or war. FCAS often have unstable and corrupt governments who are unwilling to provide basic resources and protect the human rights of their citizens [2–4]. In 2020, the World Bank classified 37 countries as fragile and conflict-affected in their annual list of FCAS.

Conflict presents as one of the world’s most significant threats to health [5]. Individuals living in FCAS suffer worse health on numerous outcomes including trauma and injuries, infectious and chronic disease, mental health, child health, and malnutrition [6]. Women, in particular, are heavily affected by ongoing conflict and violence as they obtain lower levels of education, do not have the autonomy to make decisions regarding their health, and experience abhorrent gender-based violence [7, 8]. In FCAS, women face increased barriers to accessing a continuum of sexual, productive, and maternal health services, including antenatal care (ANC). This has negative impacts on maternal mortality rates (MMR) worldwide [6]. The United Nations created Sustainable Development Goal (SDG) 3.1 in 2015 to reduce the global MMR to less than 70 per 100,000 live births by 2030 [9], from an estimated rate of 211 per 100,000 live births in 2017 [10]. Although the MMR goal outlined in SDG 3.1 is considerably lower than the current global MMR, this difference is even greater when compared to the MMR of FCAS—583 per 100,000 live births as of 2017 [11]. To work towards achieving SDG 3.1, increased attention and interventions are needed to improve maternal health service utilization in FCAS, where the MMR are highest.

ANC has been cited by numerous studies as a type of maternal health service that, if utilized, has the potential to reduce maternal mortality [12–14]. ANC is care provided to pregnant women by healthcare practitioners to identify maternal risks, prevent and manage complications, encourage positive health behaviours, and build a therapeutic patient–provider relationship [15]. In 2002, the World Health Organization (WHO) created the first set of ANC recommendations, which consisted of one first trimester visit and three subsequent visits [13]. In 2016, the WHO’s ANC recommendations increased from four total visits to eight [16]. Studies conducted prior this new recommendation in FCAS have found that the majority of women in these regions are not meeting the ANC recommendations established in 2002 [17].

**Fig. 1:**
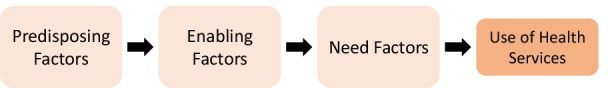
Andersen’s model of healthcare utilization (Andersen, 1995)

This systematic review is grounded in Andersen’s Model of Healthcare Utilization [18] (Fig. 1). This theoretical framework conceptualizes healthcare utilization as a function of the interaction between predisposing, enabling, and need factors that influence whether women are able to seek ANC as recommended. This model was used to create themes which were found to impact women’s ANC usage and to analyze the data extracted from included articles.

### Objectives

FCAS have been previously studied, as have the numerous health outcomes of individuals living in FCAS, including maternal health. However, the common factors that prevent women living in FCAS from accessing ANC have not been well studied. Furthermore, there is a paucity in the literature on the impact of conflict on health equity in FCAS, including the intersectional effect of gender within these situations [2]. This systematic review aims to better understand the access to maternal health services in FCAS and the factors that contribute to the inequitable gap in ANC utilization. For the purposes of this study, ANC will be defined as a visit to a healthcare practitioner to receive services, such as laboratory tests, scans, or advice regarding health behaviours, while pregnant. Visits at the time of childbirth will be excluded. Our specific objectives are to (1) identify the predisposing, enabling, and need factors which prevent and/or enable women living in FCAS from utilizing ANC according to Andersen’s Model of Healthcare Utilization [18]; and (2) identify the effects of persistent conflict on women’s access to and utilization of ANC in the 37 FCAS globally.

## Methods

**Fig. 2:**
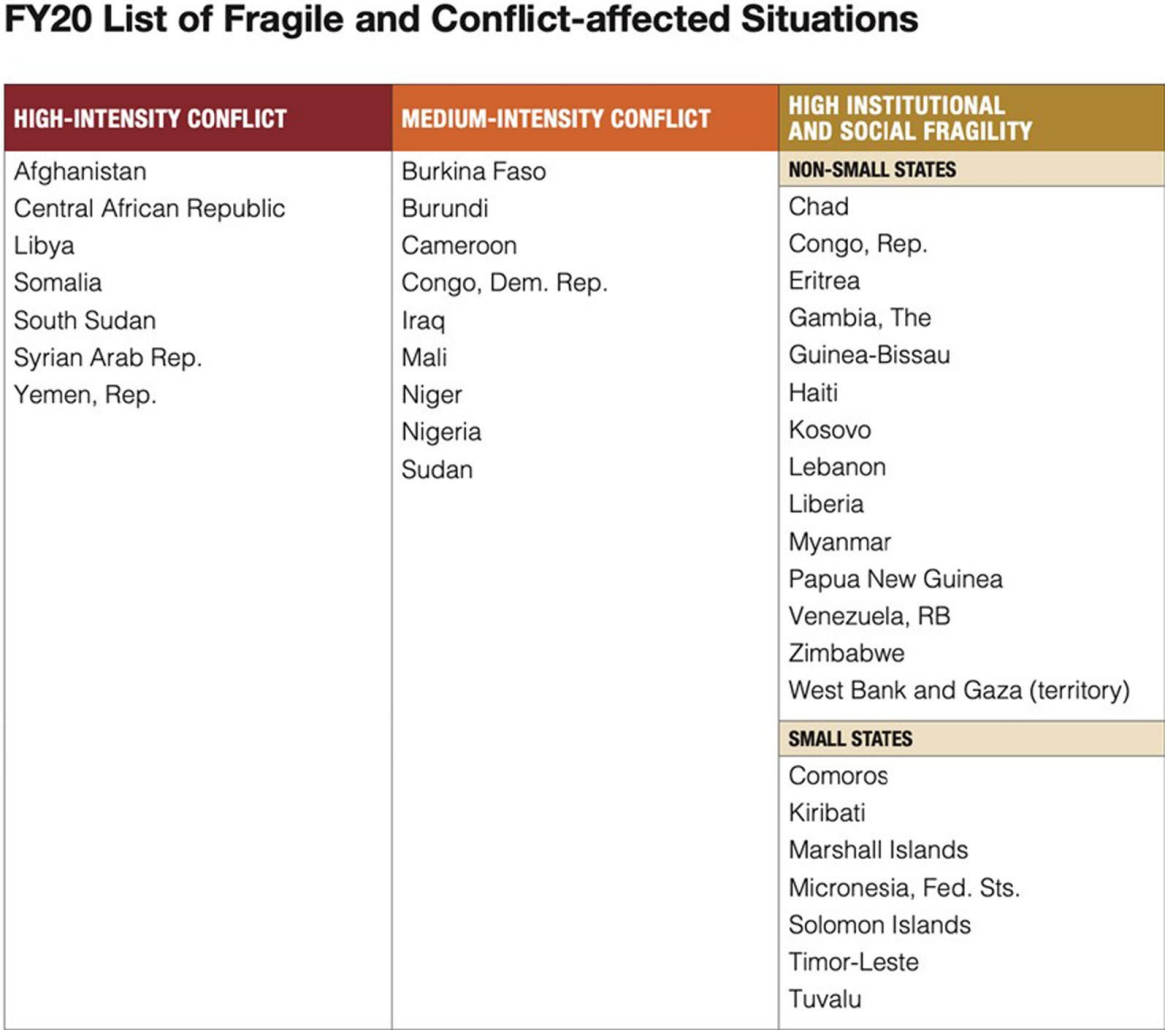
The World Bank’s 2020 list of fragile and conflict-affected situations

This systematic review was carried out to examine the barriers, facilitators, and overall factors that impact ANC usage in the 37 countries or regions classified as FCAS in 2020 by The World Bank (Fig. 2). A systematic review protocol was developed using the PRISMA checklist and uploaded to the International prospective register of systematic reviews (PROSPERO) on July 10th, 2020 (ID #: CRD42020180994).

### Search strategy

A literature search of peer-reviewed articles was conducted using SCOPUS, Web of Science, PubMed, EMBASE, and CINAHL. All five databases were searched on January 11, 2022 using a combination of MeSH terms and keywords (Table 1). The search strategy was created with the help of a subject-specific librarian and adapted to each database. Search results were limited to human studies, written in English, and published between January 2002 and January 2022.

**Table 1:** Database search terms

Population	“conflict” OR “war” OR “fragile and conflict-affected situations” OR “Afghanistan” OR “Central African Republic” OR Libya OR Somalia OR “South Sudan” OR “Syrian Arab Republic” OR “Yemen Republic” OR “ “Burkina Faso” OR Burundi OR Cameroon OR “Democratic Republic of Congo” OR Iraq OR Mali OR Niger OR Nigeria OR Sudan OR Chad OR “Republic of Congo” OR Eritrea OR “The Gambia” OR Guinea-Bissau OR Haiti OR Kosovo OR Lebanon OR Liberia OR Myanmar OR “Papua New Guinea” OR “Venezuela RB” OR Zimbabwe OR “West Bank and Gaza” OR Comoros OR Kiribati OR “Marshall Islands” OR” Federated Status of Micronesia” OR “Solomon Islands” OR Timer-Leste OR Tuvalu
Outcome	“antenatal care” OR “prenatal care” OR “maternal health services” OR “skilled birth”

All relevant studies were imported into Covidence, a web-based systematic review software, which identifies and removes duplicates, streamlines screening of citations, and facilitates the resolution of conflicts between reviewers. Two reviewers (B.Z. and K.A.) individually screened all titles, abstracts, and full texts. Disputes were resolved through general discussion with the senior author (I.L) when necessary.

### Study inclusion and exclusion criteria

Studies were eligible for inclusion if they were conducted in a conflict-affected region of one of the 37 FCAS. To achieve this, the authors identified medium and high conflict zones within each FCAS, using the Humanitarian Data Exchange or the Armed Conflict Location and Event Data Project. Any studies that took place (1) in a low conflict area of an FCAS without widespread conflict or (2) in an unspecified region of an FCAS, were excluded. Studies that utilized nationwide data, such as the Demographic and Health Surveys (DHS) and Multiple Indicator Cluster Studies (MICS), and took place in FCAS where conflict was not widespread, were excluded. This was done to ensure the data analyzed was focused on conflict-affected populations within FCAS. However, studies that utilized nationwide data were included if the FCAS had widespread conflict, such as Afghanistan. Studies published between January 2002 and January 2022 were eligible for inclusion. The year 2002 was chosen as this was when the WHO released their first set of recommendations for focused and goal-oriented ANC in an attempt to extend antenatal coverage in low- and middle-income countries [15]. Studies that identified barriers or facilitators of ANC use were included in the review. Data from women who were pregnant and had received a minimum of one ANC visit were also included in the review. Regarding study design, both quantitative and qualitative studies were eligible for inclusion. Poster presentations, conference abstracts, theses, and studies for which the full text could not be located were excluded from the review. Studies that only examined skilled birth were excluded as this type of care has been more widely studied in the context of FCAS and is not an outcome of interest in this review.

### Data extraction

Two reviewers (B.Z and K.A.) independently extracted data from all included studies. Data extracted included: list of authors, year of publication, study design, methodology employed, geographic setting, patient demographics (i.e., age, marital status), type of care provided (i.e., ANC, skilled birth), factors affecting ANC (i.e., distance, education), outcomes of interest (i.e., number of ANC visits), overall conclusions, limitations, and future recommendations. Data was extracted into a standardized extraction form developed by one of the study authors (B.Z.) using Qualtrics, an online survey platform. All bibliographic information was imported into a reference manager, Zotero, to generate citations.

### Quality assessment

Each source was critically appraised using the National Institutes of Health (NIH) Study Quality Assessment Tools [19]. The NIH tool utilized was specific to the study design of the article being reviewed. Studies were evaluated on the clarity of the research question, described eligibility criteria, choice of study population, sample size, outcomes measured, and type of statistical analysis employed. After the assessment, articles rated as either “good”, or “fair” were deemed to have high internal validity and were included in the review. Eight studies were classified as “poor” quality which would have caused them to be excluded, however, they were also excluded for other reasons including wrong geographic location. Discrepancies between reviewers were resolved through general discussion with the senior author (I.L.) when necessary.

### Data synthesis

A narrative synthesis approach was employed to analyze the data extracted from all included articles. The factors that were found to affect ANC utilization across all included studies were inductively coded [20] by two independent authors (B.Z and K.A) according to Andersen’s Model of healthcare utilization. Factors were coded as either *predisposing*, *enabling*, *need* or *other* factor type. To gain cross-study synthesis, the geographic distribution of the studies, participant demographics, and primary outcomes measured were analyzed and the percentage of women who met the ANC recommendations were calculated whenever possible. Due to the inclusion of qualitative studies and of studies with varied designs and methodologies, the data collected was heterogenous and a meta-analysis could not be carried out.

## Results

**Fig. 3:**
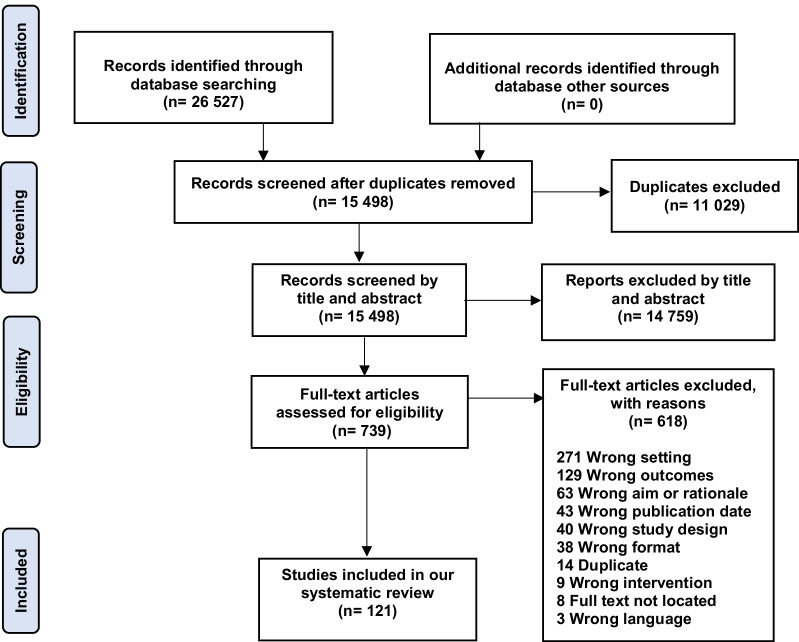
PRISMA diagram. *The total number of reasons for exclusion of the full texts exceeds 618 as some studies were excluded for multiple reasons (i.e., poor quality in addition to another factor)

The database search yielded 26,527 studies. After exclusion of 11,029 duplicate articles, and completion of title and abstract screening, a total of 739 studies were included for full text review. After applying inclusion and exclusion criteria, 121 studies were retained for inclusion in the final dataset (Fig. 3). Due to the large number of full-text studies included in this review and the heterogeneity in the designs of the included studies, a thematic description of the results is presented. A description of each article is outlined in the Additional file 1: Table S1.

**Fig. 4:**
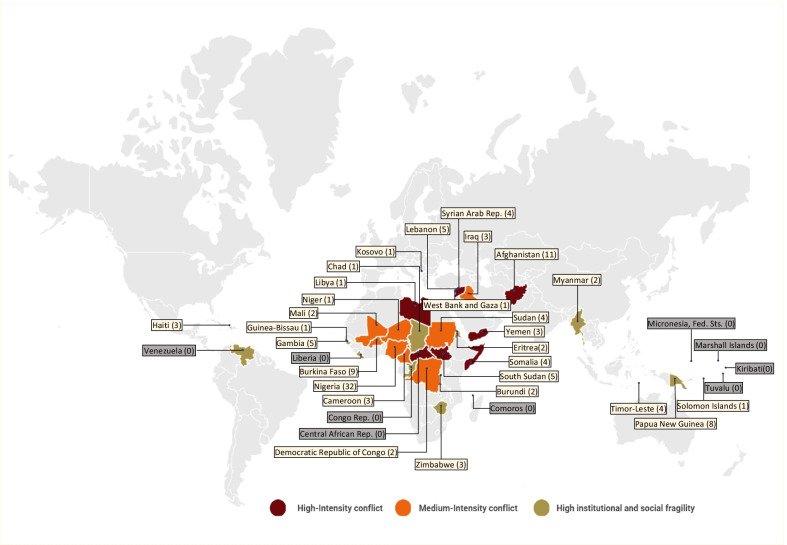
Geographic spread of articles (n = 99)

The geographical spread of the studies included in this systematic review and the number of articles per country is outlined in Fig. 4. The number of articles represented within Fig. 4 exceeds the total number of studies included as some articles examined ANC in multiple countries. Among the 121 articles included, ANC usage was examined in 123 settings: 77 articles in Africa, 15 articles in the Middle East, six articles in Southeast Asia, 11 articles in Central Asia, nine articles in Oceania, three articles in the Caribbean, one article in Palestine, and one article in Europe. Specifically, ANC was examined in 28 of the 37 regions identified as FCAS in 2020 by The World Bank. The nine FCAS for which no relevant studies were found include: Congo (Rep), Liberia, Central African Republic, Comoros, Venezuela, Kiribati, Marshall Islands, Federated States of Micronesia, and Tuvalu. Thirty-two studies analyzed utilization of care in Nigeria, which highlights that ANC has been extensively studied in this country.

**Fig. 5:**
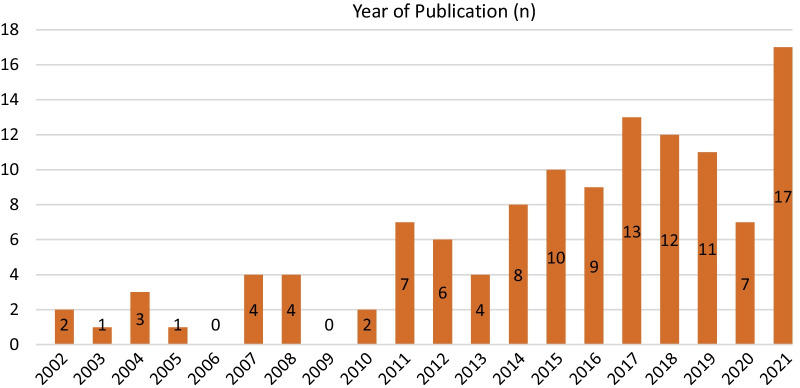
Publication year of included articles (n = 121)

The studies included were published between 2002 and 2022, with most articles being published in 2014 or later (Fig. 5). The increasing number of studies over time indicates that research on ANC has been of interest since the Millennium Development Goals and SDGs targets on maternal mortality were established in 2000 and 2015, respectively.

Overall, the studies suggest that booking the first ANC visit late in pregnancy is very common in FCAS [21–25]. Many studies also indicate that while progress has been made, women in FCAS are not meeting the WHO 2002 recommendation of four ANC visits and are therefore not meeting the 2016 recommendation of eight ANC visits [26, 27]. Table 2 identifies the factors that impact use of ANC in the 121 included articles. Seeing that many studies identified multiple factors that impact ANC utilization, the total number of factors highlighted in Table 2 exceeds 121. In accordance with Andersen’s model, *predisposing* factors include demographic, social, and contextual items such as education, employment, marital status, gender dynamics, religion, and culture [28, 29]. *Enabling* factors include financial and organizational items such as conflict, structural resources, safety, distance from ANC resources, perceived poor quality of ANC, and socioeconomic status [28, 29]. Additionally, *need* factors, which indicate a woman’s perceived need for ANC, include parity and previous complications. Finally, factors such as unwanted pregnancies, interventions (i.e., performance-based financing, home visits, mobile phone support and health education), and a husband’s education or employment were categorized as *other*. The most cited factors impacting ANC were socioeconomic status, poor quality of ANC, and education. Table 2 presents the 20 factors impacting ANC identified in the 121 included articles.

**Table 2:** Factors influencing the uptake, frequency, and timing of antenatal care

Factor type	Factor	Mentions n (%)	Article reference
Predisposing			
	Education	49 (40.5)	[17, 21, 23, 25–27, 30, 31, 33, 35, 38, 40, 41, 47, 49, 54, 60, 63, 65, 70, 71, 73, 74, 77, 106–130]
	Gender dynamics	26 (21.5)	[17, 26, 30, 42–48, 50, 54, 56–58, 60, 65, 67, 72, 76, 110, 116, 131–134]
	Culture	22 (18.1)	[22, 24, 36, 43, 46–48, 56, 57, 60, 65, 67, 68, 76, 77, 116, 121, 126, 131, 135–137]
	Region of residence (urban/rural)	15 (12.4)	[25, 32, 35, 39, 51, 57, 69, 74, 77, 109, 123, 129, 138–140]
	Marital status	14 (11.6)	[17, 33, 45–47, 54, 63, 73, 111, 117–119, 130, 131]
	Age	14 (11.6)	[25, 27, 33, 35, 36, 71, 74, 113, 121–125, 128]
	Religion	9 (7.43)	[17, 34, 37, 39, 47, 110, 123, 126, 141]
	Employment	8 (6.61)	[17, 23, 30, 33, 40, 45, 63, 71]
	Health beliefs	6 (4.96)	[22, 30, 49, 50, 77, 114]
	Ethnicity	6 (4.96)	[34, 68, 69, 77, 112, 119]
Enabling			
	Socioeconomic status	68 (56.2)	[17, 21–23, 26, 27, 30, 32, 34, 37, 38, 40, 41, 43–52, 55–58, 62, 63, 65–68, 71–74, 76–80, 105–107, 110–112, 116, 119, 122, 124, 126, 128, 130, 131, 134–138, 140, 142–147]
	Poor quality of ANC	49 (40.5)	[17, 23, 25, 27, 32, 40, 42, 43, 50, 51, 53, 55, 57–60, 62–68, 72, 76, 79, 106, 110, 111, 113, 116, 118, 126, 127, 134–137, 139, 142, 146–154]
	Distance	47 (38.8)	[17, 21, 25, 32, 34, 42–44, 47–52, 54–58, 61–63, 70, 71, 73, 74, 106, 110, 112, 113, 116, 118, 119, 121–123, 131, 134, 136, 137, 143, 147–150, 155, 156]
	Transportation	14 (11.6)	[21, 27, 38, 43, 44, 48, 50, 53–57, 61, 147]
	Infrastructure/resources	11 (9.09)	[27, 59, 61, 63, 67, 107, 134, 141, 142, 152, 154]
	Conflict	9 (7.43)	[48, 68–71, 110, 137, 146, 157]
	Safety	4 (3.31)	[48, 72, 110, 137]
Need			
	Parity	21 (17.4)	[17, 23, 25, 30, 33, 35, 40, 50, 54, 63, 70, 71, 73–76, 106, 117, 119, 124, 128]
	Previous complications	5 (4.13)	[65, 77, 106, 123, 127]
Other*		49 (40.5)	[30–32, 36, 38, 45, 47, 50, 53, 64–66, 68, 73, 74, 76–81, 102, 105, 106, 108–110, 112, 113, 120, 122, 123, 125, 127, 130, 140, 141, 143, 144, 146, 153, 154, 156, 158–163]

^*^Other factors include: Ebola, husband’s education and employment, interventions (i.e. performance-based financing), unwanted pregnancy, stigma, weather, traditional healers, media exposure, community advice, ignorance/negligence, awareness/knowledge, and contraception use

### Predisposing

#### Demographic characteristics

Demographic factors, including level of education, region of residence, marital status, age, religion, and ethnicity were cited 115 times as factors that impact ANC utilization. Education was the second most commonly cited factor that influenced ANC use in 49 studies (Table 2). Generally, women with no education or lower levels of education had decreased awareness and utilization of ANC during the first trimester and were less likely to receive the recommended number of ANC visits [30]. In contrast, women with higher levels of education were significantly more likely to book ANC early in pregnancy and to attend the recommended number of ANC visits [31]. Numerous studies also demonstrated that a husband’s level of education impacted a woman’s ANC usage [30]. It should be noted that this effect was smaller than the impact of a women’s educational attainment.

Region of residence and rurality were found to impact women’s utilization of ANC in 15 articles. The majority of studies found that, when compared to women living in rural areas, women living in urban areas within an FCAS were more likely to receive the recommended number of ANC visits and to have increased uptake of ANC overall [32].

Marital status was reported as a factor that influenced ANC utilization in 14 studies. In general, married women were more likely to use ANC as recommended compared to single women [33, 34]. Specifically, the studies found that being married increased the likelihood of early initiation of ANC [33]. The type of marital union also impacted ANC usage, where women in polygamous marriages were more likely to utilize ANC services [34].

Maternal age was shown to be a factor influencing the timing and frequency of ANC utilization in 14 studies. Most studies, with the exception of Benage et al. [27] and Bashour et al. [35], found that younger women were less likely to seek ANC early in pregnancy, receive the four recommended ANC visits, and use ANC overall [36].

Religion was reported to be a factor influencing ANC utilization in nine studies, however, its impact was context dependent. De Allegri et al. [34], found a negative association between traditional African religions and ANC uptake. Conversely, a study by Nwakamma et al. [37], found that introducing and connecting women to ANC services through faith-based communities and leaders was an important factor in promoting ANC.

Finally, an individual’s employment status was reported to be both a facilitator and barrier to ANC uptake in eight studies. Failing et al. [38], found that women’s employment negatively impacted use of ANC, where women placed more importance on completing work responsibilities to survive financially than on take time off to receiving ANC. According to other studies, using ANC four times, as previously recommended by the WHO, was generally positively associated with women’s employment [17, 39, 40]. Additionally, numerous studies found that a husband’s occupation or employment status (categorized as *other*) positively influenced women’s maternal healthcare utilization. To illustrate, Abimbola 2016 [30], found that a man’s occupation determines their wife’s socioeconomic status, which is an *enabling* factor that impacts ANC utilization [26, 38, 41].

#### Gender dynamics

Gender dynamics, which for the purposes of this study includes autonomy, decision-making abilities, and intimate partner violence, was found to impact ANC use in 26 studies. Women with higher autonomy, specifically financial autonomy, and increased decision-making abilities had greater uptake of ANC [42, 43]. Receiving permission from the husband was cited as an additional barrier to accessing ANC in numerous studies [44–46]. Furthermore, women who did not experience intimate partner violence and who did not believe that wife-beating was acceptable were more likely to use ANC and meet the recommendation of four ANC visits [17].

#### Cultural and health beliefs

Cultural and health beliefs were reported to influence ANC uptake in 22 and 6 studies, respectively (Table 2). Culture was found to shape a woman’s beliefs about ANC and pregnancy, as well as her autonomy to make healthcare decisions [22]. For example, some women believed that their baby would be in danger or that enemies would bewitch them and cause them to miscarry if the pregnancy was disclosed too early, which resulted in late initiation of ANC [22, 47]. Furthermore, in some traditions it is customary for a woman’s mother-in-law to decide whether or not she can receive care [46, 48], which can further decrease ANC utilization. Women’s health beliefs, specifically those who believed that ANC was beneficial, were more likely to use maternal health services compared to those who believed ANC was only for curative purposes. Additionally, many women believed that pregnancy is a natural process and care should only be sought if one becomes ill or develops complications [49, 50]. Therefore, the type of health belief that a woman held regarding the utility of ANC played a role in whether or not they utilized it.

### Enabling

#### Socioeconomic status

Socioeconomic status or financial difficulty was the most cited factor that prevented women from using ANC early and receiving the recommended number of visits. It was reported to influence ANC uptake in 68 of the 121 studies included in this review. The majority of studies found that women with higher socioeconomic status or wealth were more likely to utilize ANC in general, to initiate ANC early in pregnancy, and to receive the four recommended visits [26, 38, 41].

#### Distance & transport

Distance to the nearest ANC facility was the fourth most commonly cited reason for late or insufficient ANC uptake in 47 studies (Table 2). Women who lived closer to healthcare facilities or perceived the nearest healthcare facility as close to them, had higher levels of ANC usage. Unsurprisingly, those who lived further away from the nearest health facility were less likely to receive four ANC visits, initiate ANC early in their pregnancy, and use ANC overall [51, 52]. Transportation was found to be a barrier of ANC uptake in 14 studies included (Table 2). Telfer et al. found unavailability of transportation to be one of the most important barriers preventing women from accessing ANC. Pregnant women also cited having to walk to the ANC facility and having inadequate modes of transportation (i.e., rickshaws, bicycles, motorbikes) as key barriers to accessing care. The high cost of transportation was also associated with fewer ANC visits and an overall lack of ANC utilization [30, 64].

#### Poor quality of ANC

Poor Quality of ANC was reported to be a barrier to ANC uptake in 49 studies [53, 59]. Women who believed they received low quality care were less likely to meet the WHO ANC recommendations [42, 60]. Women cited lack of resources (e.g. ultrasound machines, providers etc.) [60–63], shortened hours of operation [27, 60], long wait times [64], and a lack of trust in providers [65, 66] as reasons for poor quality of care. Women also stated that healthcare providers were incompetent and had negative attitudes [43, 50], which may explain the distrust they experienced [65].

#### Infrastructure and resources

Infrastructure or lack of resources was a factor reported to impact access to ANC in 11 studies. Studies found that women who perceived operational and infrastructure problems in their community (i.e., lack of electricity, running water, destroyed building infrastructure) were deterred from accessing ANC and faced poorer health outcomes as a result [61, 67]. A study conducted by Mourtada et al. [63], found that as infrastructure destruction increased because of conflict, there was an associated decreased uptake of ANC.

#### Conflict & safety

Conflict and safety were reported as factors that directly impacted the uptake of ANC in nine and four studies [48, 68–72], respectively. Women in zones of high conflict had poorer rates of ANC utilization. Due to prolonged conflict in FCAS, women felt unsafe or insecure travelling to ANC facilities, especially alone, and were therefore less likely to seek care as recommended [48, 72]. This impact is intersectional as prolonged conflict negatively impacted education, fertility rate, availability of resources (e.g. machinery and providers), quality of care, and infrastructure, which in turn further decreased ANC utilization [48, 68, 69]. Increasing severity of conflict resulted in a decreased number of women in these areas meeting the WHO’s 2016 ANC recommendations. Finally, ANC was negatively impacted by a woman’s proximity to the conflict zone [70].

### Need

#### Parity

Parity, defined as the number of births a woman has had, was shown to be a factor that influenced ANC use in 21 studies. Women who did not have previous birth experience or who had low parity were more likely to initiate ANC early in pregnancy and to attend a greater number of ANC visits [73]. In contrast, women with higher parity were less likely to receive early ANC, attend the recommended number of visits, or meet the WHO’s ANC recommendations [17, 74, 75].

### Other

In 49 studies, women’s utilization of ANC was impacted by several *other* factors (Table 2). One commonly cited *other* factor was husband’s education and employment, where women whose partners had higher levels of education or formal employment had increased usage of ANC [38]. Unwanted pregnancies [45, 73, 76], stigma from the community or family members [36, 50, 74], community members advising against using formal ANC services [21, 77], use of traditional healers [47, 77], lack of awareness and knowledge [31, 38, 78, 79] and performance-based financing interventions [80, 81] were additional factors associated with delayed and less frequent use of ANC.

## Discussion

This review identified 20 factors that impacted ANC utilization across 28 of the 37 regions classified as fragile and conflict-affected by the World Bank in 2020. This is the first review, to our knowledge, that examines ANC utilization in FCAS, exclusively. Overall, the 121 studies included demonstrate that women in FCAS are not meeting the WHO recommendations for ANC use. When compared to women worldwide, those living in FCAS are significantly less likely to seek ANC early in pregnancy or attend a total of four ANC visits, which makes them even less likely to achieve the WHO’s 2016 recommendation of eight ANC visits [82].

Although all 121 studies examined ANC in FCAS, only nine studies (7.43%) identified conflict as a direct barrier to accessing care. We posit that while conflict was not a frequently cited barrier, it may largely explain women’s poor uptake of ANC. For example, in some FCAS, healthcare facilities are attacked, practitioners may be kidnapped, killed, or forced to flee to urban areas to ensure safety, and clinics often lack necessary resources [83]. These events may explain why women experience poor quality of ANC and cannot find care facilities in rural areas [6, 84, 85]. Furthermore, in regions of conflict, women may more often be raped by members of the militia. This leaves women less likely to seek ANC out of fear of experiencing violence when travelling to a healthcare facility alone [86, 87]. This discussion highlights the intersectional relationship between conflict and the four most cited factors impacting ANC [6, 88–90], namely education, gender dynamics, socioeconomic status, distance and quality of ANC.

Education was the most commonly cited *predisposing* factor affecting ANC utilization. Specifically, lack of education resulted in decreased utilization of ANC, which is consistent with literature on maternal healthcare utilization. In FCAS, students and teachers may be killed or displaced due to targeted attacks or recruitment initiatives by military groups [91]. This prevents schools from re-opening and decreases the number of students enrolled should schools reopen [91]. Women are often prematurely forced out of the education system to care for their family after their fathers and brothers are recruited into the military or because of unwanted pregnancies, secondary to rape. Women who are unable to obtain higher levels of education are less likely to know the benefits of ANC or the recommendations regarding timing and frequency of use [12, 82, 88, 92].

Gender dynamics, which encompasses gender-based violence and lack of autonomy, was cited 26 times as a *predisposing* factor that impacts initiation and frequency of ANC. In conflict-affected areas, the gender dynamics are strained, which puts women at higher risk of experiencing sexual violence and military sexual slavery [93, 94]. Should a woman become pregnant secondary to rape, she must ask for permission and financial support from her husband before seeking out necessary maternal care [12, 96]. Lack of autonomy to make decisions about contraception use [8, 97, 98] may also increase the likelihood of unwanted pregnancies, which is an *other* factor negatively impacting ANC use [6, 97, 99]. Women who are granted permission to seek ANC may still be unable to access it due to safety concerns associated with transport or lack of infrastructure in regions of high conflict.

Socioeconomic status, an *enabling* factor, was the most cited factor impacting ANC use. In regions of conflict, employment opportunities are limited, which makes it difficult for women to obtain the financial resources to pay the service and transportation fees associated with ANC. As a result, women may accept employment opportunities that put them at risk of physical and sexual harm, which may cause prenatal complications [95]. Should these women succeed in accessing timely and cost-effective ANC, they may not be able to afford the medications needed to ensure a healthy pregnancy. Women with lower socioeconomic status are also less likely to obtain higher levels of education, have financial autonomy, or be employed [100], which are all known to impact ANC utilization.

Distance was the fourth most commonly cited factor affecting the use of ANC. Distance is commonly thought of as the geographical space between a woman’s home and the nearest health facility [43]. In FCAS, conflict results in displacement of communities and the destruction of roads, transport vehicles and healthcare facilities, which all contribute to the increased distance between residential communities and healthcare facilities [101, 102]. Interestingly, this review found that perceived distance, which is how far a woman believes the nearest ANC facility is to her, also impacted uptake of ANC. Perceived distance is influenced by weather conditions, physical terrain, lack of transportation, and fear of travelling to healthcare facilities alone [8, 103]. Overall, distance, both real and perceived, to the nearest healthcare facility was found to impact ANC utilization and these distances may be increased in regions of conflict.

Poor quality of ANC was the second most commonly cited *enabling* factor impacting ANC uptake during pregnancy [6, 104]. Women reported experiencing long wait times and receiving care from providers who were unfriendly and “inept” [30]. Conflict directly affects resource allocation and contributes to a lack of providers, equipment, and medical resources, which may explain the poor quality of care [83]. Pregnant women in FCAS are a vulnerable population who are often unaware of the benefits of ANC [38]. When a woman feels she received poor quality ANC, it may reinforce the idea that ANC has little benefit and deter her from seeking it in the future. As such, the shortage of healthcare resources in FCAS as a result of conflict makes it difficult to provide women with high quality care which appears to have negative impacts on ANC utilization.

In order to start addressing the *predisposing* barriers that women living in FCAS face when seeking ANC, policies must be changed and region-specific interventions are needed. First, policies that prioritize girls’ access to education should be implemented to ensure they can continue with their studies if they become pregnant. Second, educational curricula should be modified to teach students the importance of using contraceptives and seeking ANC. It is also an opportunity to target cultural beliefs that claim use of ANC early in pregnancy can bewitch a child and lead to miscarriage. Third, there is a need to increase the employment opportunities for women. This will allow women to have increased financial autonomy and higher socioeconomic status, which are both positively related to ANC utilization [40]. If girls are educated and women are employed, the gender dynamics that are prevalent in FCAS may also be redefined.

To mitigate *enabling* factors, governments should provide safe and affordable transportation, cost-effective ANC services, and incentives to ANC providers. Providing transportation will help women feel safer when travelling through regions of conflict to seek ANC. Similarly, subsidizing the costs associated with ANC will help alleviate the financial burdens that women of low socioeconomic status face when seeking care. Performance-based financing schemes, which have been implemented in some FCAS [105], may financially incentivize healthcare workers to provide high quality, patient-centered ANC. It would be important, however, to ensure that a portion of the money practitioners receive is used to hire additional personnel and purchase necessary equipment, which will further ameliorate the quality of care provided.

Addressing the barriers that prevent uptake of ANC will require a grassroots approach and cooperation from several stakeholders, which may be complex, costly, and lengthy. Local policy makers, women utilizing ANC within FCAS, and global organizations, such as the WHO, should collaborate and discuss the local context, the effect of conflict on utilization of ANC, and the factors that impact its uptake. This will maximize the potential to create effective change to increase women’s access to and utilization of ANC in FCAS.

### Limitations

This review has some limitations that must be considered. First, we excluded studies not published in English, conducted prior to 2002, and for which the full text could not be accessed. Considering English is not the official language in many of the FCAS analyzed, this review may be missing relevant studies. Second, our search string was created according to the World Bank’s 2020 list of FCAS; however, studies from as early as 2002 are included in this review. As such, some of the analyzed data may have been collected at a time when the region was not classified as fragile and conflict-affected and may not represent the current barriers women in these regions are facing. Third, the included studies are heterogeneous and differ in their study design, sample size, and overall quality, which ultimately prevented us from carrying out a meta-analysis. Furthermore, many studies used self-reported data, which is subject to recall and social desirability biases. Despite these limitations, we used systematic methodologies informed by the PRISMA guidelines to conduct this review and have ensured the quality of the research findings by including studies that were rated as fair or good according to the NIH’s Quality Assessment Tools. Finally, this review does not include studies that utilized nationwide data (i.e., DHS and MICS), which may identify other factors that limit use of ANC. However, elimination of those studies was done to ensure that the data analyzed was specific to conflict-affected populations.

## Conclusion

The findings of this systematic review demonstrate that women living in FCAS worldwide face many barriers to accessing ANC. These women are not meeting the WHO 2016 recommendations of eight ANC visits, which is contributing to the high MMR in these regions. Although conflict was not commonly identified as a barrier to accessing maternal health services, it is likely that the frequently cited factors, namely socioeconomic status, distance, education, quality of ANC, and gender dynamics, are exacerbated by the effects of conflict.

### Future research

Our findings revealed that research on the factors that affect utilization of ANC is needed in the nine FCAS that are not represented in the included studies. Additionally, it is evident that the direct and indirect impacts of conflict on women’s healthcare utilization have not been well studied. Future research is urgently needed to understand how conflict impacts ANC uptake if we hope to lower the global MMR and achieve SDG 3.1 by 2030.

## Supplementary Information

**Additional file 1: Table S1.** Detailed description of included studies in this systematic review (n = 121).

## Data Availability

All data generated or analysed during this study are included in this published article and its supplementary information files.

## Abbreviations

ANC: Antenatal care
FCAS: Fragile and conflict-affected situations
MMR: Maternal mortality rate
SDG: Sustainable development goal
WHO: World Health Organization
