# Supplementary material for: Factors impacting antenatal care utilization: a systematic review of 37 fragile and conflict-affected situations
Source: Confl Health. 2022 Jun 11;16:33. doi: 10.1186/s13031-022-00459-9 (PMC9188725; doi:10.1186/s13031-022-00459-9)
Supplement: Supplementary file 1 — Additional file 1: Table S1. Detailed description of included studies in this systematic review (n = 121). [file 13031_2022_459_MOESM1_ESM.docx]

| **Supplemental Materials**  **Table 1 – Summary of Articles Included in the Review** | | | | | | |
| --- | --- | --- | --- | --- | --- | --- |
|  |  |  |  |  |  |  |
| **Authors, Years** | **Country** | **Type** | **Sample size** | **ANC Outcome** | **Barriers and Facilitators** | **Important Conclusions** |
| Abimbola et al., 2016^30^ | Nigeria | Observational Study/Cohort Study | 455 households | Awareness, knowledge, and perception of ANC | Gender dynamics (permission from husband), Education, Employment, Parity, Socioeconomic status, Traditional beliefs, Long wait times, Poor attitudes of health workers, Inadequately trained staff, literacy among husband, husband’s occupation. | Many women were aware of the benefits of ANC however, numerous barriers prevented women from seeking ANC. Husband’s education and employment had a positive impact on ANC use. |
| Adam et al., 2015^31^ | Sudan | Pre/Post | 640 internationally displaced women living in camps in Western Darfur, Sudan. | Women’s awareness regarding the existence of ANC and receiving 3 or more ANC visits | Education, Interpersonal education and mass media intervention | Awareness-raising activities carried out by community workers familiar with the socio-cultural norms and dialects spoken in the IDP camps and higher educational attainment increased the awareness and utilization of reproductive healthcare services in emergency settings in Darfur, Sudan. |
| Adebangbe et al., 2021^123^ | Nigeria | Observation study/ cohort study | 422 respondents who had a birth five years before the survey | Number of women who received one to three ANC visits and four or more ANC visit | education, distance, age, religion, rural/urban residence, camp of residence, access to radio and TV, availability of reproductive health workers, and the attitude of health providers, complications | Education, age, quality of ANC (number of healthworkers), camp of displacement and access to newspaper were significantly associated with more ANC visits |
| Akseer et al., 2016^138^ | Afghanistan | Observational study/Cohort Study | 21,290 women | ANC with a skilled provider (ANCS), 4 or more ANC visits | Socioeconomic status, Urban/Rural residence | ANC usage in Afghanistan remains low with socioeconomic status and region of residence heavily influencing ANC use. |
| Al-Mujtaba et al., 2016^33^ | Nigeria | Observational study/Cohort Study | 68 women | ANC use in a facility | Distance, Transportation, Gender dynamics, Culture, Socioeconomic status, Poor quality of ANC (poor attitude and unprofessional conduct of providers) | Distance to facility, transportation cost, healthcare provider attitude, and gender dynamics (decision-making) emerged as barriers to ANC use. |
| Alam et al., 2016^67^ | Burkina Faso | Observational study/Cohort Study | 9 interviews with midwives, nurses and women 340 women surveyed 12 focus groups | ANC usage | Distance, Gender dynamics, Socioeconomic status, Transportation, Culture | Barriers to ANC were linked to scarcity, quality of transportation, time, distance, socioeconomic status, and cultural practices. |
| Alenoghena et al., 2015^107^ | Nigeria | Observational study/Cohort Study | 342 women | Number of ANC visits | Education, Other source of information, Clean facility, Perceived health, Socioeconomic status | Most women utilized ANC as recommended. ANC use was influenced by education, sources of information, attributed of the facility, perceived health, and socioeconomic status or cost. |
| Alex et al., 2017^59^ | Haiti | Observational study/Cohort Study | 4 Haitian saj fanm, 10 matwons, and 7 postpartum mothers. | ANC use | Distance, Lack of providers, Lack of infrastructure, Lack of basic resources, Transportation | Numerous factors including distance, lack of infrastructure, transportation, human resources, and supplies barred women from attending ANC. Lack of basic resources including food also prevented women from focusing on ANC. |
| Alhaidari et al., 2017^108^ | Iraq | Pre/Post | 243 women (Control = 146, Intervention = 97) | Number of ANC visits | Education, Intervention (mobile phone support) | Mobile-phone-based pregnancy support intervention is feasible, reasonably acceptable by Iraqi pregnant women, of low cost and encourages their ANC visits. Education also impacts ANC. |
| Alosaimi et al., 2019^47^ | Yemen Rep | Observational study/Cohort Study | 7295 women | ANC adequacy, (index – timing of first visit, number of visits, and content of ANC) | Education, Socioeconomic status | ANC use in Yemen was low, higher socioeconomic status and education lead to increased ANC use. |
| Andrew et al., 2014^50^ | Papua New Guinea | Observational study/Cohort Study | 94 in-depth interviews: 52 with women, 16 with relatives of women, 12 with community leaders, and 7 with healthcare providers.  27 case studies with women. | ANC usage and timing of first ANC visit | Distance, Gender dynamics, Parity, Socioeconomic status, Poor quality of ANC, Transportation, Medication, Knowledge, Stigma, Unwanted Pregnancy, Knowledge/Awareness, Health Beliefs, Time | A number of factors facilitated and discouraged ANC attendance including those related to accessibility, attitudes, knowledge, and interpersonal factors. |
| Arnaert et al., 2019^32^ | Burkina Faso | Pre/Post | 19 women | Number of ANC visits | Distance, Gender dynamics, Poor quality of ANC | mhealth services increase access to healthcare. Distance, gender dynamics, and poor quality decreased ANC use. |
| Ashir et al., 2013^82^ | Nigeria | Pre/Post | 1,076 pregnant women | 4 ANC visits | Performance-based financing | Performance based financing lead to an increase in ANC use. |
| Badal et al., 2015^63^ | Somalia | Observational study/Cohort Study | 15 women | Knowledge of ANC, Opinions of ANC, Barriers to using ANC | Gender dynamics, Education, Culture, Socioeconomic status, Poor quality of ANC, Knowledge, Problems/complications, Time, Caretaking responsibilities for other children | With knowledge and support women can attend antenatal care. Barriers to accessing ANC may be exacerbated due to marginalization and the vulnerable lives of the participants. |
| Balinska et al., 2019^109^ | Iraq, Lebanon | Observational study/Cohort Study | 5925 women | Number of ANC visits | Education, Region, Unwanted pregnancy | The WHO targets for ANC are not feasible in these contexts where access to ANC is poor. Women with unintended pregnancies were less likely to access ANC at all drawing attention to the need for family planning. ANC use was higher in areas where NGOs including MSF had intervened. |
| Bashour et al., 2008^42^ | Syrian Arab Republic | Observational study/Cohort Study | 500 mothers | ANC use, 4 ANC visits, timing of first ANC visit | Education, Parity, Residence (urban/rural), Having an early visit, Age | Most women had more than 4 ANC visits and had a visit in the first trimester. Urban residence, lower parity, lower age, and higher education significantly increased first ANC visits. |
| Bayo et al., 2021^137^ | South Sudan | Observation study/ cohort study | 4775 (2492 maternity wards admissions were retrieved in 2015 and 2283 in 2016) | Change in number ANC visits since renewed conflict | Conflict, access to healthcare facility, autonomy in decision, culture, low perceived need, Socioeconomic status, distance, safety, poor quality of ANC | There was a decline in total ANC visits from 2016 due not only to economic and conflict-related factors, but also as a result of long term conflict and poor quality of ANC services. |
| Benage et al., 2015^27^ | Lebanon | Observational study/Cohort Study | 420 Syrian refugee women | Number of ANC visits, scope of ANC, Antenatal health practices | Education, Socioeconomic status, Poor quality of ANC, Transportation, Hours of operation, Age, Registration, Living arrangements, Lack of staff | 82.9% of pregnant women reported at least one ANC visit however only 15.7% reported 4 or more visits. In comparison, 64% of pregnant Syrian women had at least 4 ANC visits prior to the conflict. Unregistered refugee women had the lowest ANC use. Difficulty securing transport, shortened clinic hours (one third of the study period took place during Ramadan), lack of trained health personnel, and costs prevented women from accessing care. Older women (aged 35 or above) are especially vulnerable to living in insecure living arrangements and had the least frequent visits, they were also willing but unable to prevent pregnancy. |
| Chi et al., 2015^110^ | Burundi (also looked at Uganda which is not a FCAS) | Observational study/Cohort Study | 63 interviews, 8 FGDs  115 participants: 46 women of reproductive age, 32 local health providers, 37 NGO staff | Factors affecting ANC use, have these factors changed over time, the impact of conflict on ANC use | Conflict, Distance, Gender dynamics, Education, Religion, Safety, Socioeconomic status, Poor quality of ANC, Performance-based financing | Individual, socio-cultural, and political factors influence ANC use in post-conflict settings. The main factors influence ANC uptake are financial barriers, availability of support for women; distance to health services; and community perceptions of ANC. Exposure to conflict exacerbated these barriers by decreasing educational attainment and increasing the fertility rate. |
| Chittambo et al., 2003^142^ | Zimbabwe | Observational study/Cohort Study | 30 women | Factors impacted ANC usage | Socioeconomic status, Poor quality of ANC, Infrastructure | Women were not attending ANC due to fees, lack of infrastructure, and healthcare provider attitudes. |
| Chol et al., 2018^58^ | Eritrea | Observational study/Cohort Study | 40 women, 5 husbands, 10 healthcare providers, 5 decision-makers=5 | Open ended questions regarding ANC | Gender dynamics, Education, Culture, Poor quality of ANC | Health education and improvement in gender equality based on the women in Eritrea composing 33% of the armed forces lead to improved ANC use. Poor ANC quality due to lack of resources and staff prevented women from seeking ANC. |
| Christou et al., 2020^146^ | Afghanistan | Observation study/ cohort study | 55 participants (mothers and fathers who experienced a stillborn birth) | Factors impacting uptake of ANC in Afghanistan | lack of trained staff, Socioeconomic status, perceived poor quality of ANC, conflict, lack of knowledge | SES was correlated to decreased ANC uptake in Kabul, Afghanistan. Lack of knowledge and poor quality of ANC resulted in decreased ANC visits. |
| Currie et al., 2021^155^ | Afghanistan | Observation study/ cohort study | 61 maternity care providers interviewed, observed 413 ante-natal care consultations, 671 births, and 393 postnatal care consultations at public health facilities across Afghanistan | Identifying factors impacting ANC quality | infrastructure, lack of resources, poor quality of ANC | Women accessing ANC services were subjected to mistreatment and poor quality of ANC as a result of healthcare providers who work under conditions of stress with poor infrastructure and support. |
| De Allegri et al., 2011^41^ | Burkina Faso | Observational study/Cohort Study | 1050 households, 435 women | At least 3 ANC visits | Distance, Religion, Socioeconomic status, Ethnicity | Burkina Faso is making substantial progress; 97% of women had at least 1 ANC visit although a quarter did not use ANC regularly. Increased socioeconomic status was negatively associated with ANC utilization; poorer women might have benefited the most from the country’s new financing policy. Distance remained the single most important barrier in limiting access to care; religion and ethnicity were also important factors. |
| Doctor et al., 2012^49^ | Nigeria | Observational study/Cohort Study | 6,882 women | Utilization of ANC | Education, Culture, Socioeconomic status, Beliefs, Time, Region, Ethnicity, Community Advice, Traditional healers, Previous complications | ANC usage was low and is impacted by culture, money, region of residence, perception of ANC, beliefs, literacy, time, ethnicity, advice, traditional healer usage, and previous deaths of children. |
| Donmozoun et al., 2014^68^ | Burkina Faso | Observational study/Cohort Study | Total sample size n=30  30 in-depth interviews, 8 focus groups with 8-12 participants and 6 non-participant observation | Community perception of the quality of care, barriers to use of ANC | Distance, Gender dynamics, Culture, Socioeconomic status, Transportation, Poor quality of ANC, Region | Barriers to ANC: Gender dynamics/cultural barriers (lack of decision-making power to use health facility), distance to health facility and lack of transportation, financial barrier to pay for ANC and drugs, and poor quality of ANC. |
| Ebeigbe and Gharoro 2007^43^ | Nigeria | Observational study/Cohort Study | 6806 births (114 teens) | Booking status | Age, Culture (fear of telling parents), Lack of knowledge | Teenagers were more likely to be unbooked and to book late possibly due to lack of knowledge and the fear of stigma from their communities or parents of being unmarried. |
| Ebeigbe and Igberase 2005^24^ | Nigeria | Observational study/Cohort Study | 303 women | Time of first ANC visit | Culture | Most women did not have their first ANC visit in the first trimester. Socio-demographic and obstetrics factors were not significant, cultural factors should be investigated. |
| Edie et al., 2015^25^ | Cameroon | Observational study/Cohort Study | 385 women | Timing of first visit, Number of visits, Choice of site, Knowledge of ANC activities, Satisfaction | Distance, Education, Parity, Poor quality of ANC, Region (urban/rural), Referral from other women, Age | Most women did not have their first visit in the first trimester. High education, low parity, distance, and low ANC quality lead to decreased ANC usage. Referrals from other women increased ANC use. |
| Edmond et al., 2018^143^ | Afghanistan | Pre/Post | 5780 women received intervention Evaluation survey data collected from 2780 women  Data collected from 1407 eligible women (baseline), 1320 women (endline) | At least one ANC visit | Distance, Socioeconomic status, Community health worker (CHW) home visits (intervention) | The home visiting intervention improved ANC usage and knowledge, especially in rural areas. Quality of ANC should be improved to improve usage. |
| Edmond et al., 2019^144^ | Afghanistan | Observational study/Cohort Study | 1199 -Intervention baseline 1254 - Intervention endline  1242 - Control baseline 1237 - Control endline | ANC usage | Socioeconomic status, Intervention (conditional cash transfers) | Conditional cash transfers increased ANC usage in intervention groups. The effects of the intervention were lower in poorer socioeconomic status groups. |
| Edmond et al., 2020^159^ | Afghanistan | Pre/Post | There were 338 796 pregnant women in the 54 intervention and 56 control districts in 4 provinces. | At least one ANC visit | Intervention (mobile health teams) | Mobile health teams increased women’s usage of ANC by 15% including in conflict-affected and remote districts. |
| Edu et al., 2017^39^ | Nigeria | Observational study/Cohort Study | NA | Use of ANC | Distance, Socioeconomic status, Poor quality of ANC, Region (urban/rural), Lack of information | Removal of fees resulted in increased ANC usage. Women living in urban areas and living closer to clinics had higher usage. Cost of services, lack of information, and poor quality of care were identified as barriers to ANC use.  In addition to fee-removal other barriers to ANC usage must be addressed, |
| Ekabua et al., 2011^70^ | Nigeria | Observational study/Cohort Study | Observed 20 obstetric registrars, survey of 200 patients, Focus group sample size NA | Number of ANC visits, Barriers to operation of ANC | Gender dynamics, Culture, Socioeconomic status, Poor quality of ANC, Lack of facilities, Power outages | Operational and infrastructure problems prevent ANC usage. Women were unsatisfied, sought care elsewhere, and faced poor health outcomes as a result. |
| Ekzayez et al., 2021^158^ | Syria | Observation study/ cohort study | 597,215 patients coming from 11 governorates, 33 districts, 82 sub districts and 882 villages in Syria | Number of ANC visits | Exposure to armed conflict such as bombardments, explosions, and clashes was found to be a barrier to ANC uptake. | There was a negative correlation between bombardmnets and ANC visits in Syria between 2014 and 2017. |
| El-Kak et al., 2004^45^ | Lebanon | Observational study/Cohort Study | 538 women completed the first interview, 396 were followed up with | Timing of first antenatal visit, Frequency of visits, Content of ANC offered | Region | Most women had their first ANC visit in the first trimester and used a SBA for  ANC. Women in Beirut made more ANC visits than women in the Beqaa and were more likely to have higher education, be employed, be older, married later, and have lower parity. |
| Erismann et al., 2021^151^ | Chad | Cross-sectional study | 1082 women living in the Yao and Danamadji districts in Chad | Number of women who recieved at least one ANC visit and four or more ANC visits | Barriers to ANC uptake included distance to the nearest health facility, lack of familiarity towards use of ANC and being a mobile pastoralist. | Women living in Chad were less likely to utlize ANC if they were mobilize pastoralists. Barriers to accessing care include distance and familiarity with use of ANC. |
| Failing et al., 2004^44^ | Papua New Guinea | Other - case-control | 48 cases, 96 controls | Reasons for non-attendance of ANC, awareness of ANC importance | Education, Socioeconomic status, Husband’s employment , Transportation/accessibility, lack of knowledge, Time, Shame and fear | Lower education and socioeconomic status were associated with lower ANC use. Approximately half of women reported financial difficulties associated with obtaining ANC. Women who were employed did not have the time to use ANC, those who were not married and women with higher parity experienced shame, which kept them from obtaining care. Some women were also unaware of the benefits. |
| Fakunle et al., 2014^106^ | Nigeria | Observational study/Cohort Study | 33,864 women | ANC utilization | Socioeconomic status, Health insurance | This insurance scheme helped to increase the number of women attending ANC. |
| Feng et al., 2021^124^ | Nigeria | Cross-sectional study | 900 women of reproductive across eight rural communities in Delta State, Nigeria. | The influence of wealth and selected socio-demographic characteristics on antenatal care (at least 8 ANC visits) utilization. | Factors include: maternal education, age at marriage, household wealth quintile, media exposure, and parity. | Wealth is a significant predictor of ANC utilization, which suggests that affordability remains a challenge despite the free maternal care policy provided by government. Money related barriers to accesing health are especially prevalent among marginalized women living in rural places. |
| Findley et al., 2015^111^ | Nigeria | Pre/Post | 2360 from the Baseline Household Survey, 4628 in the Endline Household Survey | Improvement at endline of receiving least one ANC visit | Education, Marital status, Socioeconomic status, Poor quality of ANC | The intervention (improving ANC quality and awareness of ANC) resulted in increased likelihood of ANC use. |
| Franco et al., 2008^112^ | Mali | case-control | 817 MHO member households, 787 non-member households, 676 control households in areas without MHOs. | 4 ANC visits | Distance, Education, Socioeconomic status, Ethnicity, MHO membership, Household size | MHOs (mutual health organizations) improve ANC utilization. However, distance remains a significant negative predictor of ANC. |
| Galadanci et al., 2007^139^ | Nigeria | Observational study/Cohort Study | 2101 women | Attendance at ANC, source of ANC, number and timing of ANC, quality of ANC | Region, Poor quality of ANC | Number of ANC visits and timing in these areas of Nigeria are well below what is recommended likely due to regional differences as well as poor quality of care. |
| Gao and Kelley, 2019^113^ | Haiti | Observational study/Cohort Study | NA | Number of ANC visits | Distance, Education, Poor quality of ANC, Unwanted pregnancy, Age, Dependency | Distance and quality impacted ANC. Increased education is associated with increased ANC use. Unwanted pregnancy, dependency and age are negatively associated with ANC use. |
| Grenier et al., 2019^153^ | Nigeria | Pre/Post | 1075 women | 4+ ANC visits | Poor quality of ANC, Group-ANC | Group-ANC positively impacts ANC and quality of ANC received. |
| Gure et al., 2015^64^ | Somalia | Observational study/Cohort Study | 21 Somali women (3 focus groups with married women, 1 focus group with unmarried women) from the IDP camp. Each group had 5-6 participants. | Attitudes towards ANC | Socioeconomic status, Poor quality of ANC, Distrust of healthcare workers, Misinformation | Misinformation, restrictive laws and policies, mistrust of clinicians, and cost of services kept women from using ANC. Culturally appropriate information and services need to be identified and communicated to women. |
| Haggaz et al., 2008^48^ | Sudan | Observational study/Cohort Study | 402 women | ANC usage | Distance, Education, Socioeconomic status, Beliefs | Most women did not use ANC due to education, distance, lack of services, too expensive, and beliefs about benefits and harms. |
| Hyzam et al., 2020^53^ | Yemen | Observation study/ cohort study | 15 mothers and 9 health professionals | Thematic analysis of themes underlying use of ANC | Barriers include: cost, inadequate healthcare resources, inadquate government funding, perception of the quality of ANC, perceived need of ANC and presence of fighting near healthcare facilities, distance | The uptake of ANC of mothers in Yemen was influenced by distance, cost, perceptions of care, and presence of conflict which has decreased the quality of care. |
| Ibnouf et al., 2007^77^ | Sudan | Observational study/Cohort Study | 400 married women aged 15-49 years | ANC use - ANC monthly starting from the second trimester | Distance, Education, Socioeconomic status, Poor quality of ANC, No complications, Not available, Parity, Early stages of pregnancy | Women living in rural areas used ANC less. Accessibility, in particular walk-time to the nearest service facility was a barrier. Higher quality of ANC and higher education resulted in increased ANC usage. |
| Ibrahim et al., 2014^114^ | Libya | Observational study/Cohort Study | 300 pregnant women. | Attitude and knowledge of pregnant women toward ANC | Education, Attitudes/Beliefs | Women who had increased knowledge and positive attitudes about ANC had higher utilization rates. Unstable security in Libya may be a factor in poor ANC use. |
| Ifenne and Utoo 2012^65^ | Nigeria | Observational study/Cohort Study | 345 pregnant women attending first ANC visit | Gestational age at booking | Distance, Gender dynamics, Education, Marital status, Parity, Transportation | More than half of the women in the study booked late. Transportation, financial constraints, and the wrong perception of the right time to book for ANC resulted in late booking. Women with higher education were more likely to book early. |
| Igboanugo and Martin 2011^135^ | Nigeria | Observational study/Cohort Study | 8 women | Views of satisfactory and inadequate ANC, barriers to ANC, what promotes positive outcomes | Culture, Socioeconomic status, Poor quality of ANC | Mandatory and free ANC would increase attendance. Women advocated for knowledge about signs of complications and higher quality care. |
| Jallow et al., 2012^62^ | Gambia, The | Observational study/Cohort Study | 502 pregnant women | Perception of ANC received (willingness to come back and to recommend to others and level of satisfaction) | Poor quality of ANC, Long wait times | Women preferred private clinics due to a higher quality of care. Problems of insufficient information and ineffective communication kept women from being satisfied with care. |
| Jeremiah et al., 2012^148^ | Nigeria | Observational study/Cohort Study | 444 women | Opinion about the quality of ANC | Distance, Poor quality of ANC | Long waiting time, industrial strikes and hostile staff attitude prevented women from seeking care. Quality of ANC must be improved. |
| Jibril et al., 2018^115^ | Nigeria | Pre/Post | 120 women (experimental group), 60 women (control) | Knowledge about ANC, Accessibility of ANC | Education | Health education intervention program increased access to ANC. |
| Kane et al., 2018^131^ | South Sudan | Observational study/Cohort Study | 5 focus groups with 8 participants, 44 interviews | Themes regarding non-use of ANC | Distance, Gender dynamics, Marital status, Culture, Socioeconomic status, Social interactions | Geographical accessibility, affordability, and perceptions (need and quality of care) prevent ANC use. Social fears and fears of dignity violations may also hold women back from using ANC. Seeking ANC is a social act which entails many social interactions, in a variety of social spaces such as the neighborhood to be traversed, the waiting area of the health facility, and the care encounter setting. Depending on the local social norms, the woman’s dignity may be upheld, promoted, threatened or violated. |
| Khanal et al., 2015^26^ | Timor-Leste | Observational study/Cohort Study | 5895 mothers | Underutilization of ANC (less than 4) | Gender dynamics, Education, Socioeconomic status | About 45 % of Timorese mothers did not make the recommended four ANC visits. Women with low socioeconomic status, lower education, and with partners with lower education should be targeted. Women should be encouraged to make decision on their own health via existing community-based programs and empowering women through education would increase their utilization of ANC services. |
| Kim et al., 2021^147^ | Afghanistan | Cross-sectional study | 9712 women were included in the analytical sample | Number of individuals with at least one skilled ANC visit. | Factors impacted use of ANC include: wealth quintiles, access to transportation, distance to nearest clinic, and perceived quality of care. | Barriers to accessing ANC in Afghanistan prevent adequate uptake and consist of travel time and perceptions of quality of care. These may be mitigated by outreach interventions, adequate funding, and incentivizing programs for patients. |
| King et al., 2013^149^ | Papua New Guinea | Observational study/Cohort Study | 477 participants | Use of ANC, perceptions of the services, barriers to their use and suggestions for improvements | Distance, Poor quality of ANC | The most important issues identified by the women were improving access, providing family planning, improving the physical comfort of health facilities and changing negative staff attitudes to the mothers and to their jobs. |
| Kiruja et al., 2017^80^ | Somalia | Observational study/Cohort Study | 138 women (Maternal near-miss n = 120, Maternal death, n = 18) | Bypassed ANC | Socioeconomic status, Poor quality of ANC, Lack of time | ANC in Somaliland is a major challenge, lack of community awareness and knowledge and lack of confidence in services were major barriers. |
| Kluckow et al., 2018^23^ | Solomon Islands | Observational study/Cohort Study | 1441 women | Timing of first ANC visit | Education, Employment, Parity, Socioeconomic status, Poor quality of ANC | Late ANC booking occurred in 85% of pregnancies and was associated with increased parity and unintended pregnancy. Many pregnancies are unplanned and the majority of women book late into ANC. |
| Laing et al., 2017^37^ | Gambia, The | Observational study/Cohort Study | 25 pregnant women, 9 male partners, 13 key informants and healthcare workers | Recognition and acknowledgment of pregnancy, recognition of need for care during pregnancy and practical barriers to attendance. | Distance, Gender dynamics, Education, Marital status, Religion, Culture, Socioeconomic status, Traditional healers | Disclosure of pregnancy during the first trimester was reported to be "dangerous" due to the risk of harm from what were called "bad people" "witches" and "enemies". Such spiritual manipulation was said to cause miscarriages. This would lead to women delaying their first ANC visit. Women also used traditional healers. |
| Laing et al., 2020^125^ | The Gambia | Observation study/ cohort study | 1611 women over the age of 16 years | Trimester of first ANC visit, and total number of ANC visits | Women who were older than 30 year, nulliparous, educated, married, and with educated partners were more likely to use ANC. | Factors impacting uptake of ANC in The Gambia include age, maternal and spousal education, parity, and timing of first ANC visit. |
| Larsen et al., 2004^116^ | Papua New Guinea | Observational study/Cohort Study | 20 pregnant or parous women | Identify perceptions, beliefs, barriers and strengths relevant to the utilization of antenatal care | Distance, Gender dynamics, Education, Culture, Socioeconomic status, Poor quality of ANC  . | The attitude of healthcare workers and their perceived ill-mannered treatment of women was one of the most significant concerns raised by the women.  Another cultural issue raised by one woman was that she missed one of her visits because her relatives were angry with her and put a curse on her. |
| Lawry et al., 2017^60^ | South Sudan | Observational study/Cohort Study | 860 females who were pregnant or had children less than 5 years of age, 144 men with a wife with these characteristics. | Number of ANC visits | Distance, Socioeconomic status, Poor quality of ANC | Funding constraints, lack of skilled providers, lack of resources, distance to clinics, rainy season flooding, poor roads and no vehicles are barriers to ANC |
| Leone et al., 2019^71^ | West Bank and Gaza | Observational study/Cohort Study | 8477 women | Number of ANC visits | Conflict, Distance, Education, Parity | ANC is one of the services most affected by conflict. Locality is also important ANC may occur in higher levels in the Gaza strip compared to the West Bank  due to the relatively small distances and the absence of checkpoints within the Gaza Strip. For all Palestinians, psychological barriers to healthcare seeking derive from people’s unwillingness to go through Israeli army checkpoints and face delays, humiliation or fear of retaliation at each barrier crossing. |
| Lowe et al., 2016^132^ | Gambia, The | Observational study/Cohort Study | 50 women | Obstacles to ANC, Who decides if you can seek ANC? | Gender dynamics | Despite women’s multiple roles in the household, their positions are unfavorable. The high maternal morbidity and mortality rate in The Gambia is related to practices associated with gender inequality. |
| Maraga et al., 2011^117^ | Papua New Guinea | Observational study/Cohort Study | 391 women | Number of ANC visits (1, 2, 3, >3, or can't remember) | Education, Marital status, Parity | Level of education, marital status, and parity impacted ANC use. Women attend ANC as a means of ensuring that all is well with the pregnancy, and once it has been initiated women do not further visits. |
| Meiksin et al., 2015^133^ | Timor-Leste | Observational study/Cohort Study | 294 women, | No ANC use, ANC use, less than 4 ANC visits | Gender dynamics (Domestic Violence) | Rural women who experienced domestic violence were more likely than other rural women to have fewer than four ANC visits. |
| Merrell et al., 2020^140^ | Papua New Guinea | Cross-sectional study | 2958 women were included in the final analysis; 1368 from 2012 (mean age = 27.1; SD = 7.1) and 1590 from 2018 (mean age = 27.3; SD = 6.9 | Timing of first ANC visit, number of individuals receiving four or more ANC visits, and number of ANC services (blood pressure, urine testing etc) received | An individual's wealth, region of residence (urban vs rural), mass media exposure, and decision-making autonomy impacted use of ANC. | Utlization of ANC was greater for women living in urban settings, with exposure to mass media, and who were involved in their care decisions. The Ebola outbreak was also found to be negatively asociated with ANC uptake. |
| Mohamed-Ahmed et al., 2018^136^ | Sudan | Observational study/Cohort Study | 30 participants | Understanding, Experience, Barriers to ANC | Distance, Culture, Socioeconomic status, Poor quality of ANC | Despite increasing numbers of maternal health centres in Khartoum, Sudan, there is still underutilization of these services. Distance, cost, lack of empathy from ANC providers, lack of health promotion about ANC, and long wait times prevent women from seeking ANC. These women have suggested that a decrease in cost and an increase in empathy and availability of doctors would improve ANC use. |
| Mourtada et al., 2019^61^ | Syrian Arab Republic | Observational study/Cohort Study | 611 women (452 women in Aleppo and 159 women in Latakia) and 721 women (577 women in Aleppo and 144 women in Latakia) | Adequacy of ANC | Distance, Education, Employment, Marital status, Parity, Socioeconomic status, Poor quality of ANC, Lack of facilities, resources, and human resources | Lower education level and not experiencing current health problems were associated with lower ANC use. It appears that ANC services were more available, accessible, and acceptable to women in Latakia than women in Aleppo. |
| Mourtada et al., 2021^156^ | Syria | Qualitative exploratory study | 30 semi-structured interviews with (18–45-year-old) pregnant women from Aleppo and Latakia, Syria. | Identifying barriers to ANC uptake in Syria through interviews. | Three key barriers to ANC uptake were identified: perceived lack of need of ANC, risks associated with accessing care, and poor quality of care. | Women in Syria were less inclined to use ANC due to a lack of understanding of the benefits of the care and the lack of availability of the ANC services, which were related distance, cost, and poor experiences. |
| Mugo et al., 2018^73^ | South Sudan | Observational study/Cohort Study | 30 women and 15 men | Perceived and experienced barriers ANC | Gender dynamic, Socioeconomic status, Poor quality of ANC, Safety | This study highlighted the impact of economic and geographical accessibility, health services availability, tradition and beliefs and safety and security on access to maternal healthcare services. Removing delivery fees (bed fees) in the public health centers is essential to minimize delay associated with out of pocket fees for accessing services. Upgrading the exiting health services with the essential resources, supplies and training is also essential. |
| Mullany et al., 2008^53^ | Myanmar | Observational study/Cohort Study | 2889 households | 4+ ANC visits | Conflict, Ethnicity, Region | Lower likelihood of receiving individual ANC among those exposed to forced displacement and decreased food security, and exposures to rights violations. Considerable political, financial, and human resources are necessary to improve access to maternal healthcare in these communities. Ethnicity and region of residence impacted whether women met the recommendation for number of ANC visits. |
| Mullany et al., 2010^103^ | Myanmar | Pre/Post | 5331 women | Use of ANC | Intervention (MOM intervention) | Access to ANC visits were substantially higher following implementation of the MOM pilot project. |
| Mutowo et al., 2021^141^ | Zimbabwe | Qualitative exploratory study | Eight community members (men, chiefs, councillors and politicians), eight five maternal healthcare providers (midwives and village health workers) and postnatal women involved in the provision and use of ANC services took part in focus group discussions | Perceived barriers to utlizing ANC services. | Barriers to accessing ANC in rural Zimbabwe included disrespect for maternal healthcare users, lack of resources at health facility, user insufficient knowledge, fear of HIV testing, cost, household re- sponsibilities, lack of spousal support and involvement and failure to integrate traditional and religious knowledge in healthcare practices. | Several barriers limit utilization of ANC services in Zimbabwe that are multifactorial in origin and include: systemic barriers related to the healthcare system, support system related barriers, cultural related barriers and religious related barriers. |
| Ndidi and Oseremen 2010^22^ | Nigeria | Observational study/Cohort Study | 348 women | Timing of first ANC visit, when is the best time for women to start ANC? | Culture, Socioeconomic status, Values, Beliefs | 3/4 of the study registered for ANC in the second trimester of pregnancy while 1/3 did in the third trimester. Reasons cited for late booking include: culture, money, and values.  Nearly a tenth of the population registered late because they wanted to delay making the pregnancy public or were afraid of perceived enemies who may harm the pregnancy. |
| Nie et al., 2016^160^ | Timor-Leste | Observational study/Cohort Study | In total, 581 women aged 15-49 with a child up to 24 months | 4+ ANC visits | Mobile phone ownership | Women who owned a mobile phone were more likely to utilize ANC. When adjusting for socioeconomic status, mobile phone ownership was not an independent predictor. Socioeconomic factors may be important barriers to the success of mHealth programs that focus exclusively on women having access to a mobile phone. |
| Ntambue et al., 2012^78^ | Congo, Dem. Rep. | Observational study/Cohort Study | 1762 women | ANC visits | Parity, Culture, Socioeconomic status, Unwanted pregnancy, Gender dynamics, Poor quality of ANC  . | Women giving birth for the first time, those who have already given birth many times, and women with an un- wanted pregnancy, made less use of ANC. The lack of information on pregnancy management may explain why first-time mothers do not use ANC.  Poor perceptions of care and not being allowed by church or husband prevented ANC use. |
| Ntambue et al., 2016^40^ | Congo, Dem. Rep. | Observational study/Cohort Study | 2394 women | Number of ANC visits | Education, Employment, Marital status, Parity, Age | Being unmarried was associated with lower ANC use. Working at home was higher in the group with high attendance. Women who had high attendance were more highly educated than those who did not receive ANC. |
| Ntoimo et al., 2019^134^ | Nigeria | Observational study/Cohort Study | There were 183 participants, 106 male and 77 females | Preferences, beliefs and perceptions about ANC, Where women access ANC,  Why they do not use ANC, Ways to improve access to ANC | Distance, Gender dynamics, Socioeconomic status, Poor quality of ANC, Lack of infrastructure, Misunderstanding of pregnancy | Rural women will not use ANC if they cannot physically access the health facilities, if the ANC centers offer low quality and non-respectful care if the cost is not affordable and partner support is lacking or minimal. |
| Nwakamma et al., 2019^51^ | Nigeria | Pre/Post | 8 key informant interviews  8 focus group discussions with congregational and community members  8 in-depth interviews with 12 religious leaders  242 pregnant women enrolled into the learning sessions | Factors that contributed to the success of the model and sustainability of CHESS-Advocates model. | Religion, Socioeconomic status | A key success of the initiative was the ability to identify and link pregnant women into ANC services through mentoring and learning sessions delivered in the faith congregations and the faith leaders’ confrontation of the gender norms that limit women’s ability to make decisions. This study has shown the promising efficacy of engaging faith communities as important factors in promoting ANC and gender justice particularly in rural and underserved communities. |
| O'Keefe et al., 2011^161^ | Papua New Guinea | Observational study/Cohort Study | Over 33 000 monthly reports | Number of women attending their first ANC visit | Village health volunteers (VHVs) impacting ANC | Found a significant increase in ANC provided by the VHVs in East Sepik Province; in the period 2007 to 2010 the proportion of all pregnant women receiving their first antenatal visit from VHVs has more than doubled. |
| Onalu et al., 2021^126^ | Nigeria | Qualitative study | 45 respondents including 21 health care professionals and 24 mothers residing in the area | Barriers to utilization of maternal health care services in the Okrika local government area of Rivers State, Nigeria | Poor income, ignorance, educational attainment absence of social support, religion and culture, inaccessibility of health centers and health care system inefficiencies (staff absenteesim) | TBAs can play a major role in increasing the utility of MHC services through adequate training in modern medicinal practices |
| Okonofua et al., 2017^56^ | Nigeria | Observational study/Cohort Study | 5 Focus groups (8-12 pregnant women each) | Women’s opinions of ANC | Poor quality of ANC, Transportation, Knowledge | Lack of information prevents women from seeking early care, difficulties they experience during transportation to hospital, and inadequacies in the healthcare delivery system (most prominent). Women were dissatisfied with the quality of care they received, poor staff attitude, long waiting time, poor attention to women in labor, high cost of services and inadequate facilities were the major reasons for dissatisfaction. |
| Okonofua et al., 2018^118^ | Nigeria | Observational study/Cohort Study | 1408 women (701 from Esan SE, 707 from Etsako East) | Place of antenatal care | Distance, Education, Marital status, Poor quality of ANC | Reasons for ANC choice include facility near to place of residence, good quality service, cost not too much, and husband wanted it. Reasons for not using ANC include: poor quality service, no provider in the facility, facility not open, facility too far, and costs too much. Considerations for distance and costs, and perceptions relating to poor quality care are the factors that mostly hinder women’s access to ANC. |
| Omer et al., 2014^17^ | Nigeria | Observational study/Cohort Study | 7870 women in Bauchi and 7759 in Cross River | 4+ ANC visits to a government health facility | Distance, Education, Marital status, Religion, Socioeconomic status, Poor quality of ANC, Gender Dynamics (Intimate Partner Violence), Parity, Employment | Low level of use of government ANC. Efforts to increase use of ANC need to focus on poor, uneducated women in rural areas. Not experiencing intimate partner violence during last year or last pregnancy, naming a government health facility as their nearest source of antenatal care, and coming from a less poor household (having enough food during last week) were associated with ANC. For Bauchi additional factors included: being gainfully employed, having more than two previous pregnancies, being from a community with a good access road and from a community with an active village development committee. |
| Othman et al., 2017^74^ | Yemen Rep | Observational study/Cohort Study | 460 | Number of ANC visits, Timing of visits, | Distance, Education, Marital status, Parity, Socioeconomic status, Absence of a health problem, Unwanted pregnancy | The findings of this study indicated that factors affecting significant utilization of ANC services were mother education, residence place, age at first pregnancy, gravida, parity, occurrence of pregnancy without planning, and number of live children. Factors affecting significant number of visits were mother education, residence place, and husband work. The main reason for not receiving any ANC and also for not returning to health facility after first visit was absence of health problems during last pregnancy. |
| Ouédraogo et al., 2021^163^ | Niger | Quasi-experimental descriptive study | 1736 women who reported attending ANC during their current pregnancy | Maternal and household characteristics, and out-of-pocket costs (OPC) , total time spent and the opportunity cost of time for ANC visits affect ANC attendance. | Women with no OPCs, in households where the household heads identified themselves as farmers and among those who were interviewed in the study during the lean and rainy seasons had lower ANC scores | Related OPCs still exist in many forms for pregnant women despite government interventions. OPCs, however, were associated with ANC attendance score. |
| Oyetundeo and Elery 2014^46^ | Nigeria | Observational study/Cohort Study | 230 women | Use of ANC (timing & number of visits) and perception of ANC | Education, Employment, Parity, Socioeconomic status, Poor quality of ANC, Perceived importance of ANC | ANC was utilized in this setting despite the participants low socioeconomic state. The major factors that influence the use of ANC were husbands’ and wives’ education level and occupation, cost of care and attitude of health workers. There is a need to empower women to attain financial freedom. |
| Oyewale and Mavundla 2015^75^ | Nigeria | Observational study/Cohort Study | 385 women | ANC use | Distance, Education, Parity, Age, Socioeconomic status, Parental consent, Stigma from early marriage or sexual coercion, Discrimination from health workers, Region | Women who were older, more educated, with higher birth order, or resident in urban areas, have a higher likelihood of utilizing ANC. |
| Oyovwe and Woolhead, 2021^127^ | Nigeria | Qualitative exploratory study | 13 in-depth interviews from seven women and six health care professionals | Barriers to the uptake of maternal health services, from the perspectives of both women and health care professionals | Mobility related costs, unfriendly health staff, subpar hospital conditions (bad odours, lack of meals and privacy), Caesarian Section induced trauma, readilty available and cheaper TBAs relative to HCPs, education, Poor personal health awareness | Barriers to the uptake of maternal health services is multifaceted, including new tangible and intangibal hurdlesl such as pregnant's womens' ignorance of the differences between TBAs and HCPs |
| Perry et al., 2007^150^ | Haiti | Observational study/Cohort Study | 50 pregnant women | Utilization of ANC | Distance, Terrain | Health needs are greater in the mountainous areas, and the inputs required to achieve the same health outcomes in the mountainous areas are much greater not only because of the greater health needs but because the difficulties of program operations are much greater (as a result of the difficult terrain, the greater dispersion of the population, the lower level of education and socio-economic status of the population, and the higher levels of childhood malnutrition). |
| Rahmani and Brekke 2013^66^ | Afghanistan | Observational study/Cohort Study | 27 people (12 pregnant women or women who had recently given birth, 7 doctors, 5 midwives and 3 traditional birth attendants) | Barriers to ANC | Distance, Socioeconomic status, Poor quality of ANC, Family dynamics, Transportation | Participants reported several obstacles to ANC including their own personal views and beliefs, family decisions, financial reasons, as well as transportation difficulties. |
| Rossier et al., 2014^119^ | Burkina Faso | Observational study/Cohort Study | 3346 live births in Nairobi, Ouagadougou 2501 births | Number of ANC visits | Distance, Education, Marital status, Parity, Socioeconomic status, Ethnicity | Maternal health in urban Burkina Faso benefits from a set of relatively well-enforced regulations and policies, including subsidized low-cost delivery, free ANC, regulations stipulating that women need at least one ANC to deliver in a hospital, and the prohibition of TBAs from assisting deliveries. |
| Rudasingwa et al., 2017^145^ | Burundi | Pre/Post | 274 women | ANC use (at least 1 ANC visit, 3+ ANC visits, ANC in hospitals or health centres), Possession of an ANC card | Socioeconomic status | There was no effect on ANC visits, which already at the baseline were at a higher level, and with relatively lower unit bonuses. |
| Samiah et al., 2021^164^ | Afghanistan | Cross-sectional study | 420 pregnant women | Factors associated with late antenatal care (ANC) initiation among pregnant women attending a comprehensive health clinic in Kandahar Province of Afghanistan | No knowledge on when to start ANC, unintended pregnancy and no ANC visit in the past | Crucial factors associated with late ANC initiation in pregnant women include: no knowledge on when to start ANC, unintended pregnancy and the absence of previous ANC visit. Hence, optimization of timely ANC initiation in the context of Afghanistan would require multimodal approaches that are culturally appropriate |
| Shabila et al., 2014^57^ | Iraq | Observational study/Cohort Study | 38 women | Agree/Disagree with 39 statements reflecting different aspects of ANC | Poor quality of ANC, Preference for Private Care, Infrastructure | Different types of problems and concerns related mainly to inadequate provision of information and poor interpersonal communication, poor utilization of public services and a general preference to use private services were reported by the different groups of women. Infrastructure and the available facilities, quality of services, qualifications and experiences of healthcare providers and/or specific experiences, expectations and characteristics of women impacted ANC. |
| Sibanda et al., 2018^35^ | Zimbabwe | Observational study/Cohort Study | 299 women | ANC use , Timing of first ANC visit | Marital status, Socioeconomic status, HIV, Anxiety due to exclusion, Nurses attitudes towards women, Partner opposition, Gender dynamics (lack of financial autonomy), Wait times, Employment (lack of time), Unwanted pregnancy | ANC uptake is sub-optimal particularly in poor communities where ANC fees are levied. Interventions to increase ANC uptake in the context of PMTCT need to address general ANC barriers and those related to the fear of HIV testing. Anxiety due to exclusion-for women, not having paid the required ANC registration fees meant exclusion from the clinic. Women reported feeling helpless and “distressed” by this exclusion. The fear of HIV testing was frequently discussed as a barrier. Nurse attitudes - as for fear of HIV testing, the role of identity creation in participant accounts was apparent: seven women reported that they knew other women who had not sought ANC because they wanted to avoid the clinic nurses who were reported to be discourteous towards clients. Nurses were also feared not to uphold confidentiality of a woman’s HIV positive status. Unsupportive male partners- Eight women reported that they had faced partner opposition to ANC registration. Because most women were financially dependent on their husbands, they did not have the power to decide when they would register. Three reasons for the lack of male support were given: first, men did not appreciate the importance of the package of ANC. Second, the man’s assertion of authority reportedly affected ANC registration negatively. Thirdly, the fear of their wife undergoing HIV testing reportedly played a role in men’s attitudes towards ANC registration.  Long waiting times at the clinic, women in self or formal employment found it hard to take time off work, unintended pregnancies, and emotional distress dur to divorce were reasons for avoiding ANC. |
| Solanke et al., 2018^72^ | Nigeria | Observational study/Cohort Study | 2729 women of reproductive age | ANC use (Less than 4 visits, 4 or more visits) | Distance, Education, Employment, Socioeconomic status, Residence type, Parity, Age, conflict | The main factors influencing ANC usage in Nigeria after Boko Haram are education, women's working status, marital status. Other factors include perceived distance to the nearest healthcare facility, poverty level, religion, maternal age, and community media exposure. |
| Somé et al., 2013^36^ | Burkina Faso | Observational study/Cohort Study | 30 women | Factors influencing health facility usage | Gender dynamics, Marital status, Culture, Socioeconomic status | This study has shown that the decision to use obstetric care in rural Burkina Faso is mainly made by men and mothers-in law, therefore, any initiatives to increase the use of clinical obstetric care should involve men, specifically husbands and partners and the woman’s mother in-law. |
| Somé et al., 2020^128^ | Burkina Faso | Observational/cohort study (secondary analysis) | 704 women of reproductive age who permanently reside on Kaya Health and Demographic Surveillance System area | Estimate the extent of delayed antenatal visits after the first trimester, and to identify its main associated factors | Age, education level of the woman, socioeconomic level of the household and parity | High prevalence of late ANC1 exist in Kaya health district and attention to factors including health education, community implication and free healthcare for mother and childmay help reduce this prevalence |
| Stanikazi et al., 2021^129^ | Afghanistan | Cross-sectional study | 850 women with at least one delivery in the last 2 years in Kandahar city | The magnitude and determinants of antenatal care services’ utilization in Kandahar city | Factors influencing uptake include: education, planning of last pregnancy, and district of residence. | Even though more than half the respondents used ANC service at least once, only about 1 in 5 utilized the recommended (4 or more) number of ANC visits. Determining factors include educational status of the mother, pregnancy intention, and place of residence. |
| Steenland et al., 2017^81^ | Burkina Faso | Pre/Post | 186 health facilities | Number of antenatal care visits, First trimester ANC visit | Socioeconomic status, Performance based financing | This study makes a strong case that PBF can be used to increase the provision of at least some targeted health services. |
| Stojanovski et al., 2017^52^ | Kosovo | Observational study/Cohort Study | 603 women | 4+ ANC visits, Quality of ANC | Conflict, Culture, Socioeconomic status, Poor quality of ANC, Ethnicity, Language barriers, Changing government, Discrimination | Women from Roma, Ashkali, and Balkan Egyptian communities in Kosovo receive poorer care as compared to Kosovar Albanian and Kosovar Serbian women. The main factors impacting this are cultural stigmas, language barriers, financial problems resulting partially from statelessness due to lack of government documentation, displacement and where the services are received. |
| Sui et al., 2021^130^ | Nigeria | Cross-sectional study | 819 women within the reproductive ages across eight rural communities in Delta State, Southern part of Nigeria. | Effect of household socioeconomic status and other socio-demographic characteristics on antenatal care (ANC: minimum of 4 and 8) utilization | 4 ANC visits: maternal education, mass media exposure, travel time to the nearest health center, cost of maternal care, mode of healthcare payments and household wealth quintile 8 ANC visits: maternal education, mass media exposure, cost of maternal care as barriers to maternal care, household wealth quintile, and marital status | Although ANC coverage is genrally low, ≥ 4 and ≥ 8 ANC utilization are determined by varying factors that will need be intimately considered in policymaking. At the primary and secondary education level, free tuition policy should be used to encourage female enrolment rate, and at tertiary level special cut off should be used to encourage female education. |
| Talhouk et al., 2016^54^ | Lebanon | Observational study/Cohort Study | 59 refugees in rural Lebanon | Influences on ANC use, Insights into ANC Experiences, Behaviors and Beliefs, Impact of technology on ANC | Distance, Socioeconomic status, Poor quality of ANC, Appointment reminder system, Region (urban/rural) | Main factors hindering Syrian refugees who were living in Lebanon's access to ANC were distance from the PHC, financial constraints, lack of an appointment system, access to transport, and having negative interactions with the healthcare workers during the visit. |
| Teguete et al., 2012^76^ | Mali | Observational study/Cohort Study | 19 253 births | The primary outcome was maternal mortality, ANC was also considered | Parity | Grand multiparas (more than 5) women are less likely to receive ANC when compared to pauciparas (1-4). |
| Telfer et al., 2002^34^ | Gambia, The | Observational study/Cohort Study | 623 women | ANC use, Number of ANC visits, Timing of first visit | Distance, Transportation. Gender dynamics, Socioeconomic status | Transportation, money/emergency costs and approval/permission from spouses are the main factors that pose a challenge to accessing ANC. Another interesting point is that many times women would miss the appointment because of an illness, which is counterintuitive. |
| Tolefac et al., 2017^55^ | Cameroon | Observational study/Cohort Study | 293 women | First ANC visit less than or equal to 12 weeks gestation or first ANC after 12 weeks gestation | Distance, Socioeconomic status, Large family size (equal to or greater than 4 ppl), | The study showed that nearly half of women starts their first ANC booking later than the WHO recommended time. Financial constraints and long distances to the hospital were major reasons advanced by about a third of those who presented late during the first ANC. Leaving more than 10 km away from the hospital, having a family size greater than or equal to 4 persons and having a lower monthly income less than 200 US dollars were factors significantly associated with late first antenatal care booking.  Thosefrom rural places were more likely to be the ones walking further and accessing ANC later than usual.  44.0%) of them came for their first ANC visit late, after 12 weeks of gestation |
| Truppa et al., 2019^79^ | Lebanon | Observational study/Cohort Study | 1179 participants | Difference in ANC usage between Lebanese and Syrian Refugees | Socioeconomic status, Lack of awareness | A stronger outreach component is needed to address lack of awareness and cost barriers must be addressed. |
| Turan et al., 2011^120^ | Eritrea | Pre/Post | 844 women | Use of ANC, Timing of first ANC visit, total number of ANC visits (4+ ANC visits) | Education, Maternal Health Volunteer Intervention | This community intervention successfully increased safe motherhood behaviors. Women in the intervention area gained knowledge about birth danger signs, increased their birth preparedness, increased their use of ANC services. The intervention resulted in more use of any ANC, more total visits during pregnancy, and first visits earlier in pregnancy. |
| Udenigwe et al., 2021^157^ | Nigeria | Qualitative exploratory study | 13 stakeholders and key informants who are policy-makers and clinical managers in Edo State. Participants include: one senior official with the State Ministry of Health, one senior official with the State Primary Healthcare Development Agency, two senior officials responsible for PHC at each LGAs, two senior LGA officials, and seven clinical managers | Policy-makers and clinical managers’ views on maternal health service delivery in rural Nigeria | Disparities in healthcare access and utilisation of various services induced by factors such as out-of- pocket costs for services and the physical accessibility of PHC facilities, lack of infrastructure and quality service, and lack of cohesive and efficient management of health services in PHC centres | Even though primary healthcare facilities provide a range of services, they operate within a selective approach to PHC. Participants generally depicted maternal care services in PHCs as acceptable yet inaccessible due to undue barriers of cost and geographic location and poor quality. |
| Vail, 2002^121^ | Papua New Guinea | Observational study/Cohort Study | 2560 women | Use of ANC | Distance, Education, Culture, Age | In Tari there is a pattern of high utilization of ANC and delivery services, but little use of family planning. The reasons for this pattern lie in the geographical and cultural setting in which the utilization of these services occurs. Among the 122 women who did not attend clinics, half said the clinics were too far away; the majority of women who gave this response lived in areas more than 2 hours from a health institution. |
| Viswanathan et al., 2012^122^ | Afghanistan | Observational study/Cohort Study | 8281 women | ANC use (at least 1 visit) | Distance, Education, Socioeconomic status, Age, Number of married women in the household, Presence of Community Healthcare Workers | Distance, presence of community health workers, wealth and formal education, age, and number of married women in the household are factors that influence the use of ANC. |
| Wallace et al., 2018^69^ | Timor-Leste | Observational study/Cohort Study | 17 women | Reasons for delays in seeking care, Delays in reaching care, Delays in receiving care, Delays from perceptions of respectful care | Distance, Gender dynamics, Socioeconomic status, Poor quality of ANC, Poor service/treatment by healthcare staff | The main factors that were identified to hinder Timor-Leste women's access to ANC were money for transport and financial costs they incur in getting to the healthcare facility. Another barrier identified was infrastructure constraints. |
| Warri and George 2020^21^ | Cameroon | Observational study/Cohort Study | 18 pregnant women and 3 key informant midwives | Perceived barriers to ANC | Distance, Education, Socioeconomic status, Accessibility, Transportation | In Cameroon only approximately 20.5% of pregnant women initiate ANC within the first trimester of pregnancy. The study showed that pregnant women and midwives have the same opinions of the reasons for the late initiation of antenatal care: - Distance (poor road network, uncomfortable transportation), Accessibility of ANC. High cost of initiating ANC, lack of information, misinformation from family members and spouses are also barriers to ANC. Some participants perceive the booking system to be user-unfriendly and complain of overcrowded conditions, long waiting times and rude clinic staff. |
| Wilunda et al., 2017^38^ | South Sudan | Observational study/Cohort Study | 127 women in the villages and 42 women in cattle camps | Access and resource availability, influence of the sociocultural context and insecurity, perceptions of pregnancy, perceptions of the quality of care | Conflict, Distance, Gender dynamics, Safety, Culture, Socioeconomic status, Transportation | In South Sudan, many factors hinder a woman's ability to access ANC including transportation problems and insecurity. In this setting, geographical barriers and insecurity made it difficult and dangerous for women to travel to health facilities. Two key sociocultural barriers to ANC utilization: women’s domestic chores and the influence of male partners/husbands. Tradition and culture influenced institutional childbirth in this setting but seemed to have a relatively weaker influence on ANC attendance. Lack of correct and sufficient information about the importance of ANC and poor understanding of the risks aggravates the socio-cultural barriers mentioned above. |
| Ye et al., 2016^162^ | Burkina Faso | Pre/Post | 23 pregnant women | 1 ANC visit, 4 ANC visits | Mobile phones | The contribution of mHealth in the access to health information and to improve population health is undeniable. In the context of a high penetration of mobile telephones within communities, it offers as an opportunity for improving health outcomes in many different ways. There was an 8% increase of ANC uptake and better compliance of HIV patients to antiretroviral services. |
